# Supplementary material for: Efficient Inhibition of HIV Using CRISPR/Cas13d Nuclease System
Source: Viruses. 2021 Sep 16;13(9):1850. doi: 10.3390/v13091850 (PMC8473377; doi:10.3390/v13091850)
Supplement: Supplementary file 1 [file viruses-13-01850-s001.zip › viruses-1329477-supplementary.pdf]

Table S1. Oligonucleotide primers

Primer	Sequence
**pBR43IeG-iRFP670-nef+ cloning primers**	
iRFPNcoIF1	taataccatggcgcgtaaggtcgatct
iRFPXmaIR1	aatacccgggttagcgttggtggtgggcggc
**pBR43IeG-iRFP670-nef+ colony PCR and sequencing primers**	
BRNL43seqF	acgttgtgagttggatagttgt
BRNL43seqR	gtactccggatgggatatcttg
**pKLV2-U6-CasRx-(pre-gRNA)-PGKpuro2ABFP vector cloning primers**	
XR4MluIF1	gccctacgcgtgagggcctatttcccatgattcctt
XR4BamHiR1	gaattggatccgcgtaaggagaaaataccgcatcag
**pKLV2-U6-CasRx-(pre-gRNA)-PGKpuro2ABFP vector sequencing primers**	
MSCrev	cagcggggctgctaaagcgcatgc
pKLV-cpptseqF1	cagtgcaggggaaagaatag
**qPCR primers**	
qCasRxF2	cagggagaaggccaaaaccg
qCasRxR2	caggtggacggccttgtttc

Table S2. guide RNA design

gRNA	HIV Genome Target Location	Gene	% Conservation in Clade B	% Conservation in All HIV-1 clades	Target 22 nt	gRNA score	Quartile	Off-targets 0:1:2:3 mismatches
BR09	1817	Gag,p24	79.9	78.3	TAGAAGAAATGATGACAGCATG	0.24	1	0:0:0:0
BR18	2328	Pol,prot	77.9	77.7	ACAGGAGCAGATGATACAGTAT	0.78	4	0:0:0:0
BR25	2329	Pol,prot	77.8	77.7	CAGGAGCAGATGATACAGTATT	0.92	4	0:0:0:0
BR24	2330	Pol,prot	75.4	75.8	AGGAGCAGATGATACAGTATTA	0.85	4	0:0:0:0
BR23	2331	Pol,prot	75.7	76.9	GGAGCAGATGATACAGTATTAG	0.85	4	0:0:0:0
BR02	2332	Pol,prot	75.4	75.7	GAGCAGATGATACAGTATTAGA	0.74	3	0:0:0:0
BR26	2333	Pol,prot	74.5	74.4	AGCAGATGATACAGTATTAGAA	0.72	3	0:0:0:0
BR21	2334	Pol,prot	76.6	75.2	GCAGATGATACAGTATTAGAAG	0.87	4	0:0:0:0
BR33	4750	Pol,int	80.7	76.1	CAGCAGTACAAATGGCAGTATT	0.74	3	0:0:0:0
BR34	4751	Pol,int	76.9	72.9	AGCAGTACAAATGGCAGTATTC	0.77	4	0:0:0:0
BR43	4753	Pol,int	77.3	73.3	CAGTACAAATGGCAGTATTCAT	0.60	3	0:0:0:0
BR29	4792	Pol,int,cPPT	67.2	71.6	AAGGGGGGATTGGGGGGTACAG	0.83	4	0:0:0:0
BR20	4794	Pol,int,cPPT	68.2	74.5	GGGGGGATTGGGGGGTACAGTG	0.80	4	0:0:0:0
BR22	4887	Pol,int,CTS	80	76.9	ATTCAAAATTTTCGGGTTTATT	0.07	1	0:0:0:0
BR19	4888	Pol,int,CTS	82.9	78.4	TTCAAAATTTTCGGGTTTATTA	0.07	1	0:0:0:0
BR27	4889	Pol,int,CTS	83	78.3	TCAAAATTTTCGGGTTTATTAC	0.08	1	0:0:0:0
BR16	4953	Pol,int	77.4	77.6	CTCTGGAAAGGTGAAGGGGCAG	0.53	3	0:0:0:0
BR17	4955	Pol,int	76.8	73.8	CTGGAAAGGTGAAGGGGCAGTA	0.49	3	0:0:0:0
BR41	4959	Pol,int	88.2	72.8	AAAGGTGAAGGGGCAGTAGTAA	0.54	3	0:0:0:0
BR04	4962	Pol,int	86.2	71.9	GGTGAAGGGGCAGTAGTAATAC	0.72	3	0:0:0:0
NT	-	-	-	-	TCACCAGAAGCGTACCATACTC	-	-	0:0:0:0

Table S3. Variation in gRNA seed sequence

gRNA	Alignment	% variation in all HIV-1 clades
BR04	gRNA seq 3'C**CACTTCC**CCGTCATCATTATG 5'	
	Consensus HIV-1 seq 5'GGTGAAGGGGCAGTAGTAATAC 3'	
	GGTGAAGG**N**GCAGTAGTAATAC*	4.9%
	GGTGAAGGGGC**N**GTAGTAATAC*	3.5%
	GGTGAAGGGGCAGT**G**GTAATAC	4.9%
	GGTGAAGGGGCAGTAGT**C**ATAC	7.7%
	GGTGAAGGGGCAGTAGT**G**ATAC	4.5%
BR23	gRNA seq 3'C**CTCGTCT**ACTATGTCATAATC 5'	
	Consensus HIV-1 seq 5'GGAGCAGATGATACAGTATTAG 3'	
	GG**G**GCAGATGATACAGTATTAG	5.0%
	GGAGCAGATGA**T**ACAGTATTAG	3.8%
	GGAGCAGATGATACAGT**G**TTAG	3.8%
	GGAGCAGATGATACAGT**C**TTAG	3.0%
	GGAGCAGATGATACAGTATT**N**G*	2.5%
BR29	gRNA seq 3'T**TCCCCCC**TAACCCCCCATGTC 5'	
	Consensus HIV-1 seq 5'AAGGGGGGATTGGGGGGTACAG 3'	
	AAGGGGGGATTGGGG**N**GTACAG*	4.9%
	AAGGGGGGATTGGGGG**N**TACAG*	11.6%
	AAGGGGGGATTGGGGGGTA**N**AG*	3.3%
	AAGGGGGGATTGGGGGGTACA**N***	3.5%
BR34	gRNA seq 3'T**CGTCATG**TTTACCGTCATAAG 5'	
	Consensus HIV-1 seq 5'AGCAGTACAAATGGCAGTATTC 3'	
	AGCAGT**G**CAAATGGCAGTATTC	4.5%
	AGCAGTACA**G**ATGGCAGTATTC	9.9%
	AGCAGTACAAATGGCAGT**A**TTC	3.4%
	AGCAGTACAAATGGCAGTA**T**TC	2.3%

*N= multiple nucleotide substitution; gRNA seed sequence is in bold blue font, gRNA-seed sequence binding-region in HIV sequence in red font, mutations in the circulating HIV-1 sequences are bold and underlined.

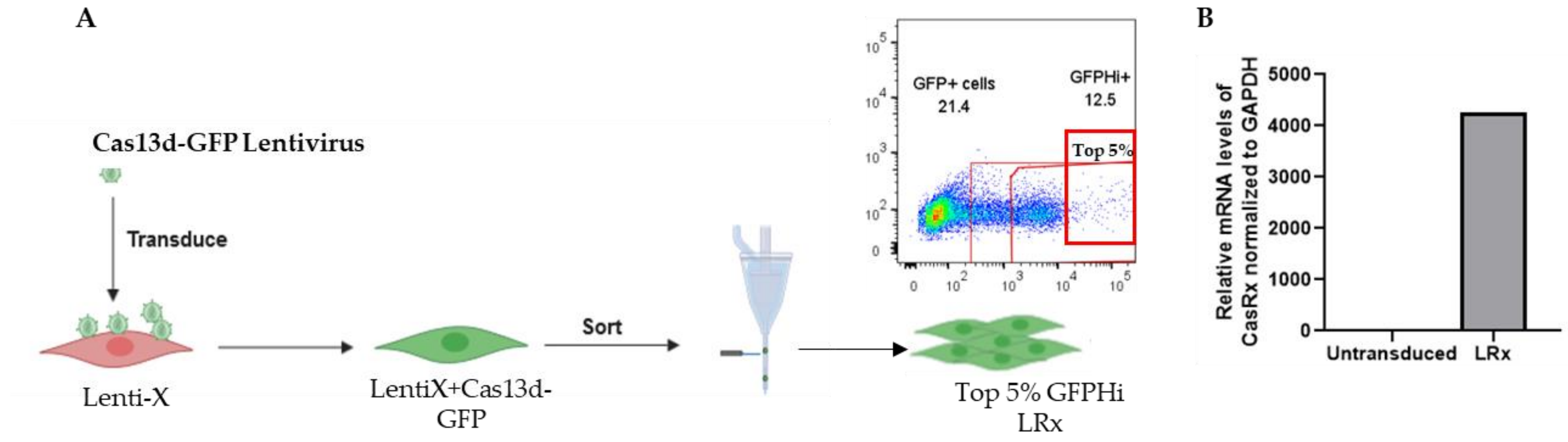

Supplementary Figure S1 (A) Gaiting strategy Lenti-X™ cell line was transduced with Cas13d-GFP lentiviral particle. Over twenty one percent cells expressed GFP (GFP+), 12.5% cells expressed high levels of GFP (GFPHi+). Cells expressing highest levels of GFP Cells expressing highest levels of GFP (Top5%) were sorted by FACS and further expanded in vitro culture. These cells with stable expression of Cas13d-GFP are termed LRx cells. (**B)** Expression of CasRx in LRx cells is confirmed by quantitative PCR (qPCR).

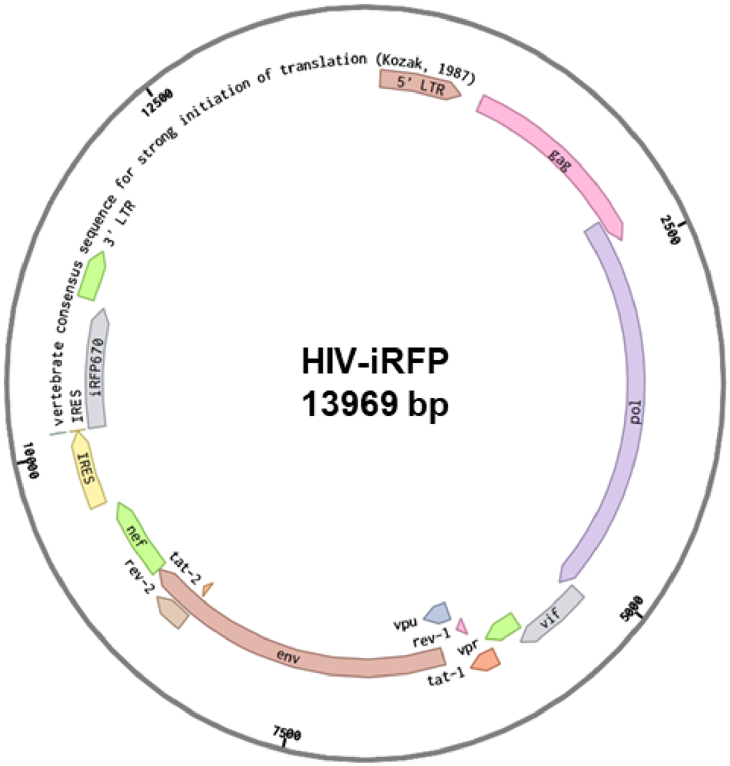

Supplementary Figure S2 Plasmid design for expression of HIV-1 encoding fluorescent tag. A full-length, chimeric and replication competent subtype B, CXCR4-tropic HIV-1 vector (dpBR43IeG-nef+ clone # 11349; NIH-AIDS reagent, which co-express eGFP and nef from a single bicistronic RNA was modified to replace eGFP by a near infra-red fluorescent protein iRFP670. This vector henceforth referred to as HIV-iRFP.

A

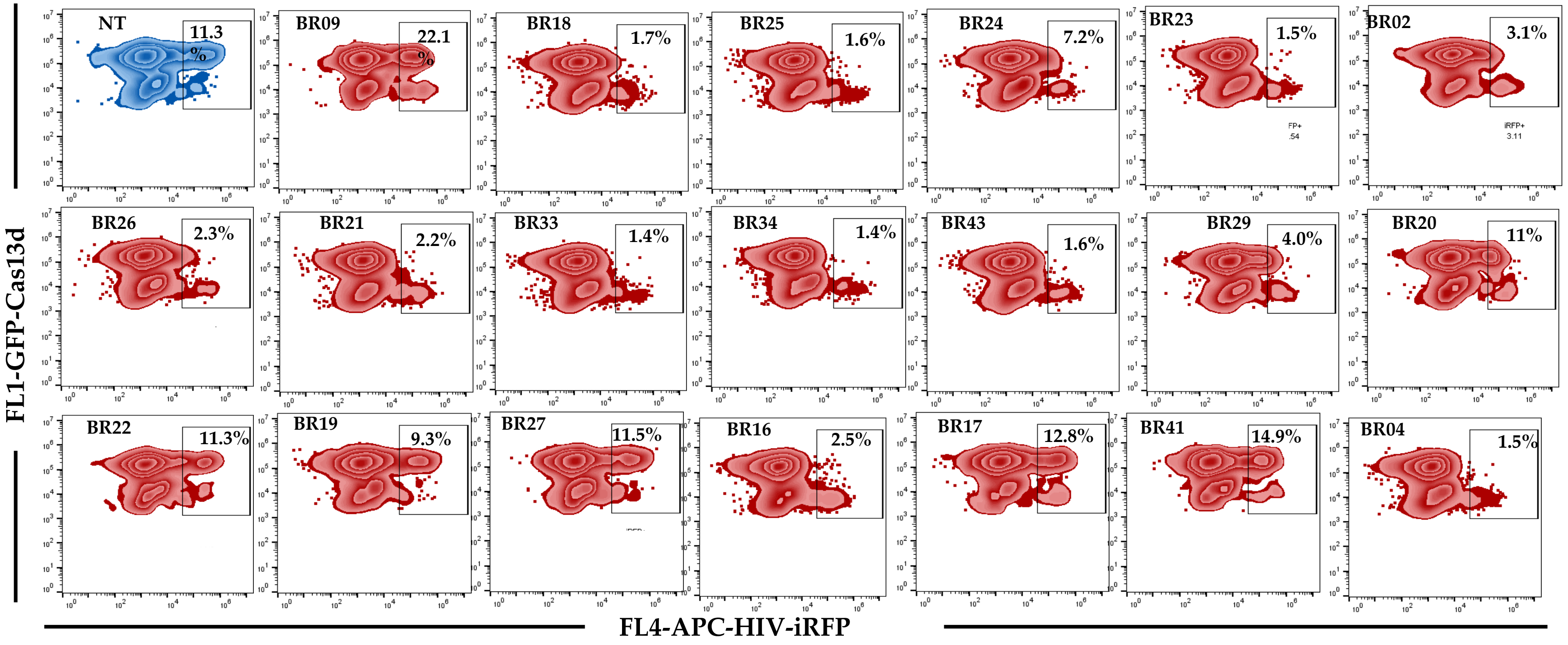
NT
11.3 %
BR09
22.1 %
BR18
1.7%
BR25
1.6%
BR24
7.2%
BR23
1.5%
FP+
.54
BR02
3.1%
iRFP+
3.11
BR26
2.3%
BR21
2.2%
BR33
1.4%
BR34
1.4%
BR43
1.6%
BR29
4.0%
BR20
11%
BR22
11.3%
BR19
9.3%
BR27
11.5%
BR16
2.5%
BR17
12.8%
BR41
14.9%
BR04
1.5%
FL1-GFP-Cas13d
FL4-APC-HIV-iRFP

B

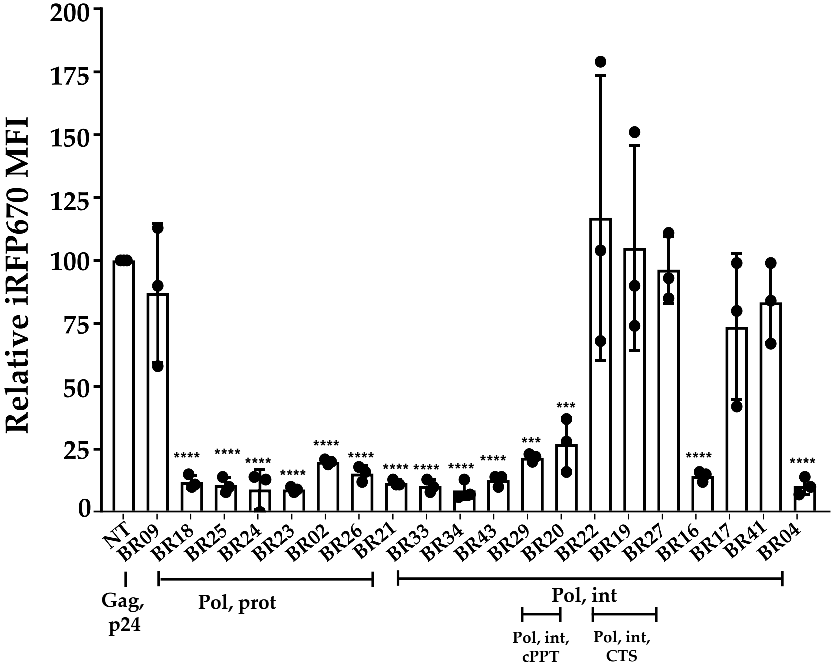

Supplementary Figure S3. **Measurement of viral replication by flow cytometry**. **(A)** Lenti-X^{TM} cells with stable expression of CasRx-GFP (LRx) were transfected with plasmids encoding molecular clone of HIV co-expressing iRPF-670 fluorescent tag (HIV-iRFP) and HIV-specific guide RNAs (HIV-gRNA, red) or a non-targeting control (NT, blue). Forty eight hours after infection, expression of CasRx-GFP and HIV-iRFP were measured by flow cytometry. The cells transfected with HIV-targeting gRNA (red) inhibited HIV as compared to the cells transfected with Non-targeting gRNA (NT, blue). **(B)** The cells transfected with HIV-targeting gRNA showed significant reduction in iRFP-670 mean fluorescent intensity (MFI) as compared to the cells transfected with non-targeting gRNA. The means ± s.e.m. are depicted as horizontal and vertical bars for each group, respectively. ANOVA tests with Bonferroni correction were used for statistical comparisons, and two-tailed *p* values are indicated. ****<0.0001; ***p<0.001, **p<0.01.

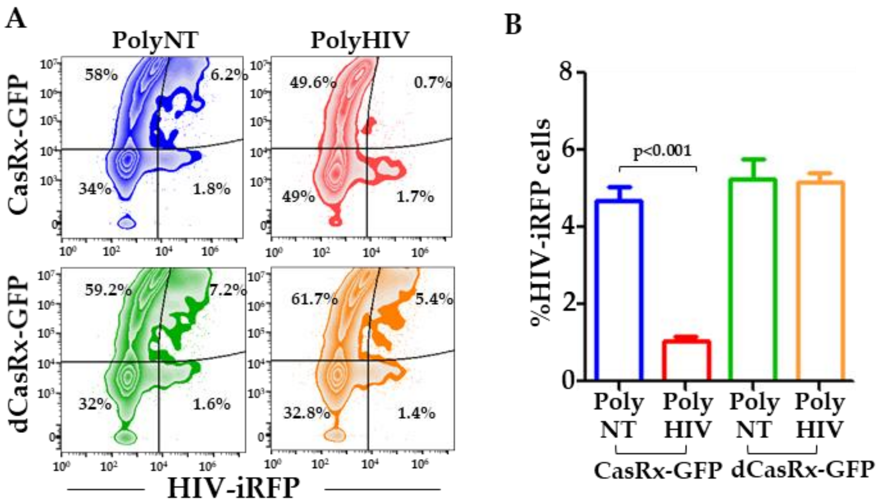

Supplementary Figure S4. **Catalytically inactive CasRx (dCasRx) with HIV-specific guide RNAs does not affect HIV replication**. (**A**) Lenti-X^{TM} cells were co-transfected with plasmid encoding CasRx-GFP or dCasRx-GFP, guide RNAs (gRNA) and HIV-iRFP. Forty eight hours after infection, expression of CasRx-GFP/dCasRx-GFP and HIV-iRFP were measured by flow cytometry. (**B)** In the cells expressing CasRx-GFP and polyHIV (red) the percentage of HIV- iRFP+ cells were significantly reduced compared to CasRx-GFP and polyNT (blue). However, percentage of HIV-iRFP cells were similar in dCasRx-GFP and polyHIV (green) and dCasRx-GFP polyNT (orange) transfected cells. The means ± s.e.m. are depicted as horizontal and vertical bars for each group, respectively. Student's t-test was used for statistical comparisons, and two-tailed *p* values are indicated.

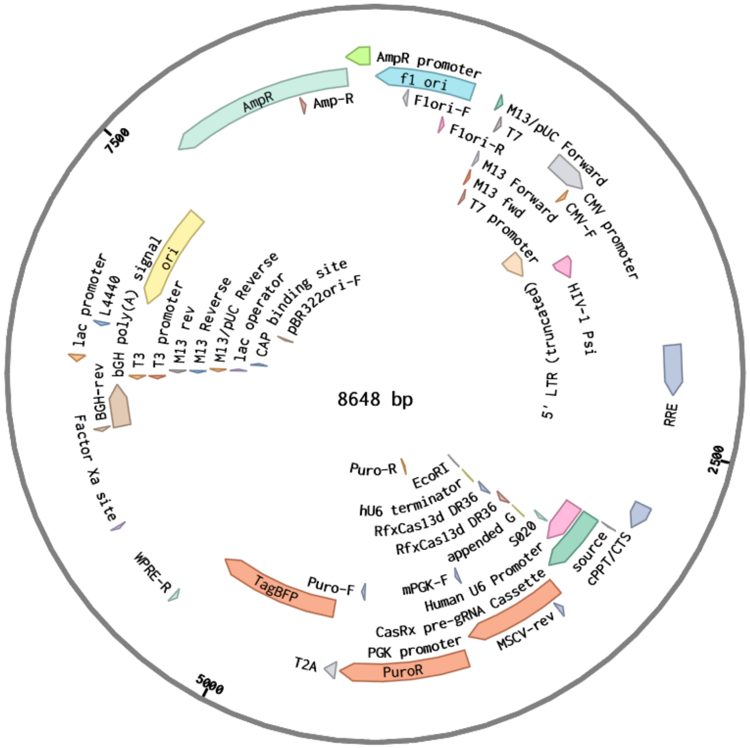

Supplementary Figure S5 Plasmid Design – pKLV2-U6-CasRx-(pre-gRNA)-PGKpuro2ABFP-W.
